# Case report on intervention of sedative drug dependence with “simple and quick reconstruction method” in psychological crisis assistance

**DOI:** 10.1097/MD.0000000000033691

**Published:** 2023-04-28

**Authors:** Yi-qun Liang, Bao-dong Yu

**Affiliations:** a Qingdao Women and Children’s Hospital Affiliated to Qingdao University, Qingdao, China.

**Keywords:** case report, COVID-19, psychological crisis assistance, psychosomatic medicine, sedation drug dependence, simple and quick reconstruction method

## Abstract

**Introduction:**

In the past, the problem of sedation drug dependence has been reported in psychological counseling cases, but it is rare to use the rapid reconstruction method for psychological emergency intervention. This article reports the applying of rapid reconstruction method in the intervention of sedation drug dependence during psychological emergency in the context of the Corona Virus Disease-2019 public health events.

**The main therapeutic interventions:**

Firstly present the problem including presenting experiences related to psychological stress, troubles of events, core problems and self-assessment based on a score of 0 to 10, secondly transfer information including normalizing relevant reactions and providing useful knowledge, thirdly cope with strategies of seeking internal and external resources, reassessing and making improvement plans, at last improve the summary which includes reviewing the process to summarize gains, initiate actions and commit.

**Outcomes:**

The author discussed the current psychological crisis with the patient, scored the tense and anxious situation, normalized the response to the patient and passed on the knowledge about controlling Corona Virus Disease-2019 prevention and sedative drugs, helped him find the method to adjust himself and the social resources he confided to his friends during the similar period, scored again, put forward the plan, reviewed the conversation process and made a commitment to not use sedative drugs.

**Conclusions:**

Through the “simple and quick reconstruction method,” the patient was able to solve the problem of dependence on sedative drugs, relieve tension and anxiety, find resources, and keep living.

## 1. Introduction

**Figure 1.**
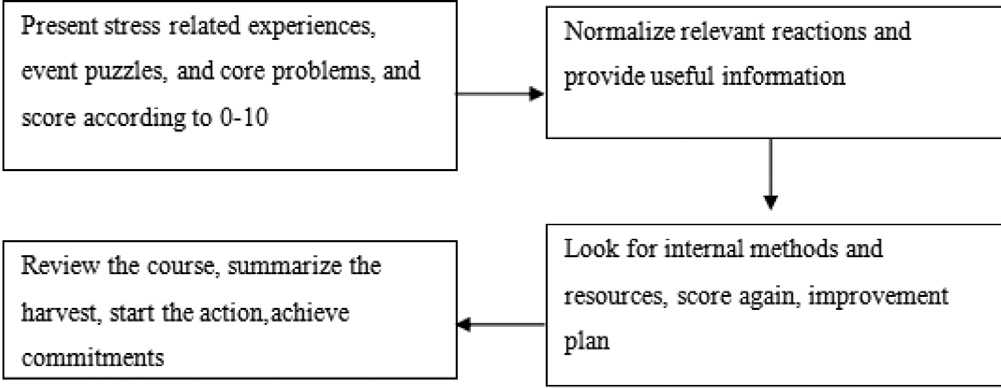
The 4 steps of “the simple and quick reconstruction method”.

Psychological crisis assistance, including understanding the psychological reaction of crisis events, seeking and establishing social support resources, and learning positive coping methods, is to give psychological first aid to the patient, increase the “tolerance window” of the patient, help them deal with urgent problems, avoid self injury or hurting others, reduce confusion and unstable emotions, reduce physical symptoms, restore adaptive social and psychological functions, and safely pass through the psychological crisis.^[1]^ The “simple and quick reconstruction method” provides a brief method in psychological assistance. Through presenting problems, transmitting information, coping with discussion, summarizing and improving, hope can be obtained and reconstruction can be promoted (Fig. 1).^[2–4]^

## 2. Informed consent

At the beginning of this psychological hotline process, the author obtained the oral informed consent of the patient: “This psychological crisis assistance is for the public good. There is no recording, and we will keep it confidential. No private information will be disclosed to the outside world. Of course, if there is suicide or self injury, juvenile abuse, Corona Virus Disease-2019 (COVID-19) infection without medical treatment or threat to the public, it will be reported to the relevant departments.” The patient agreed.

## 3. Patient consent statement

The written informed consent for inclusion of clinical details was obtained from the patient for the purpose of publication. The private information, such as name, would be anonymous when published.

## 4. Patient information

The patient was a medical staff. Since the outbreak of COVID-19, he had been worried that he would be infected with COVID-19 in the process of handling biological samples. There were 2 children at home and he were also worried that he could infect their children. For this reason, he often lost sleep, so he injected a sedative drug named propofol to relieve pressure. Recently, the epidemic had been repeated, he felt more and more nervous and anxious. The patient had not sought psychological assistance.

## 5. Diagnostic assessment

The author judged that the patient had the problem of psychological stress and drug abuse. The patient needed to evaluate himself, communicate, think about similar experiences and feelings in the past and make commitments in the process of psychological assistance, if the patient was willing to cooperate with the author.

## 6. Therapeutic intervention

The study was approved by – “Ethics Committee of Qingdao Women and Children’s Hospital. The study is in accordance with relevant guidelines and regulation.

In the psychological first aid used “the simple and quick reconstruction method” and lasted 20 minutes. Its process was as follows.

First, the author presented the stress and problem. The author turned the real problem into a psychological problem: “After hearing so much from you, you felt nervous, anxious, could not sleep well, worried that you and your children were infected with viruses, and you used sedatives. This interview could not solve all the above problems in just 20 minutes. If only one of them could be solved today, which problem would you most like to solve?” The patient answered with anxiety. Then, the author asked the patient to rate the situation on a scale of 0 to 10 points. Zero points for no trouble and neutral state, and 10 points for the most serious state imaginable. The patient answered with 10 points.

The second step is to normalize the response of the patient and pass on relevant scientific knowledge: “There are indeed new cases of COVID-19 every day. In fact, you can also learn about the psychological behavior of people around the epidemic. This tension and anxiety is a normal response and a need for survival.” Then, the author shared her experience in medical work: she also worried that she might be infected with COVID-19, and could not sleep well when she was under great pressure, and passed on the knowledge of epidemic prevention and control and the harm of sedative drug abuse to the patients: as long as he wore N95 masks according to the hospital’s protection requirements, wore and took off isolation clothing, and disinfected his hands, he could effectively avoid virus infection. Meanwhile, propofol is an intravenous anesthetic, which is drug dependent and can cause a series of adverse consequences. It has an obvious effect of inhibiting the respiratory and circulatory systems. It can only be used by trained anesthetists in clinical practice. In addition, life monitoring and emergency support systems should be available at the scene and should not be used at home.^[5,6]^

In the third step, the author first help the patient find his past experience, then his relatives, friends, leaders and other resources, as well as his future. The author asked: “Did you have any anxiety problems in the past, but you solved them later. How did you come back then?” The patient answered that he went to the gym to exercise, and fell asleep when he was tired. Then, the author asked the patient to think: “If someone can help you at this moment, who do you want to be and how?” The patient replied that he hoped to tell his friends all of this. In the above process of seeking internal methods and external resources, the author always pay attention to maintaining a firm tone and intonation, and becoming the dependence of the patient. Then, the author asked the patient to rate again: “We discussed a lot, and we also saw that you actually had similar times. After doing sports adjustment, you also found your friends who can help you. Can we rate again now? Do you remember the score at the beginning, and what do you think it is now?” The patient answered with 4 points. The author asked the patient what he planned to do as the first step, so that he could give an accessible answer: “In order to deal with the current epidemic, 4 points is also needed. What are your plans to continue your life in the future?” The patient replied that he would run during the off duty time.

In the last step, the author asked the patient to review the course, helped him summarize and improve, judged whether the patient’s ideas were biased, strengthened the positive part, so that the patient could see the solution, reach a commitment, enhance the ability of action, and make arrangements for the follow-up life: “We have talked for 15 minutes, and there are still 5 minutes left for this interview to end. Now I would like to invite you to review the whole process. Do you have any gains or insights?” The patient replied that he found a way to help himself and promised not to use sedatives any more.

Through the use of the “simple and fast reconstruction method,” the patient was helped to obtain sufficient psychological support and see hope.

## 7. Discussion

This case report described a brief reconstruction method of psychological first aid that can relieve the stress and drug dependence of the patient in a short time. This form of psychological hotline is equivalent to bandaging on the battlefield, rather than further treatment. Therefore, it is not possible to dig vertically into the emotions and feelings of the patient in the process of psychological counseling. Meanwhile, the author could not see the details like the face or expression of the speaker in the interview.

## Author contributions

**Conceptualization:** Yi-qun Liang.

Formal analysis: Yi-qun Liang.

Investigation: Yi-qun Liang.

Project administration: Yi-qun Liang.

Resources: Bao-dong Yu.

Validation: Bao-dong Yu.

Writing – original draft: Yi-qun Liang.

Writing – review & editing: Bao-dong Yu.
